# Action on the Surface: Entomopathogenic Fungi* versus* the Insect Cuticle

**DOI:** 10.3390/insects4030357

**Published:** 2013-07-16

**Authors:** Almudena Ortiz-Urquiza, Nemat O. Keyhani

**Affiliations:** Department of Microbiology and Cell Science, University of Florida, Gainesville, FL 32611, USA; E-Mail: almudenaortiz@ufl.edu

**Keywords:** insect epicuticle, entomopathogenic fungi, virulence, host defense, co‑evolution

## Abstract

Infections mediated by broad host range entomopathogenic fungi represent seminal observations that led to one of the first germ theories of disease and are a classic example of a co-evolutionary arms race between a pathogen and target hosts. These fungi are able to parasitize susceptible hosts via direct penetration of the cuticle with the initial and potentially determining interaction occurring between the fungal spore and the insect epicuticle. Entomogenous fungi have evolved mechanisms for adhesion and recognition of host surface cues that help direct an adaptive response that includes the production of: (a) hydrolytic, assimilatory, and/or detoxifying enzymes including lipase/esterases, catalases, cytochrome P450s, proteases, and chitinases; (b) specialized infectious structures, e.g., appressoria or penetrant tubes; and (c) secondary and other metabolites that facilitate infection. Aside from immune responses, insects have evolved a number of mechanisms to keep pathogens at bay that include: (a) the production of (epi) cuticular antimicrobial lipids, proteins, and metabolites; (b) shedding of the cuticle during development; and (c) behavioral-environmental adaptations such as induced fever, burrowing, and grooming, as well as potentially enlisting the help of other microbes, all intended to stop the pathogen before it can breach the cuticle. Virulence and host-defense can be considered to be under constant reciprocal selective pressure, and the action on the surface likely contributes to phenomena such as strain variation, host range, and the increased virulence often noted once a (low) virulent strain is “passaged” through an insect host. Since the cuticle represents the first point of contact and barrier between the fungus and the insect, the “action on the surface” may represent the defining interactions that ultimately can lead either to successful mycosis by the pathogen or successful defense by the host. Knowledge concerning the molecular mechanisms underlying this interaction can shed light on the ecology and evolution of virulence and can be used for rational design strategies at increasing the effectiveness of entomopathogenic fungi for pest control in field applications.

## 1. Introduction

Microbial pathogens of insects have evolved a wide range of strategies for overcoming the formidable defense systems that insects, in turn, continue to evolve in order to fend off such attempts at infection. The selective pressures on the pathogen and the target host have led to a co-evolutionary arms race that is increasingly being recognized as having led to some startling outcomes. Modern exploration of entomopathogenic fungi began with the work of Agostino Bassi (1773–1856), whose observations on the fungal-mediated *mal de segno* or white muscardine disease of silkworms led to one of the first germ theories of disease [1]. This review will focus on advances in examining the interactions of broad-host range entomopathogenic fungi with the insect (epi)-cuticle, limiting consideration to a subset of insect pathogens in which such interactions are an integral part of the infection process. From a teleological perspective, keeping pathogens at bay at the outermost surface (*i.e.*, epidermis or in the case of insects, the cuticle or epicuticle) would seem to be preferable since it would maximize the probability of survival while minimizing the metabolic costs of a defense response and the chances for an infection to spread throughout the organism. Although certainly a vital component of an insect’s defense response, limiting pathogens on the surface, would imply that the immune system might represent more of a second and ultimately final effort at fighting infection of certain pathogens (or at least prolonging survival to reproduction) than the cuticular defenses. Thus, the insect cuticle along with behavioral avoidance, exclusion or removal of infected nest-mates, especially in social insects, likely represent the two boundary defenses that shape and determine the outcomes of interactions between entomopathogenic fungi and target hosts. However, whereas the immune system has garnered much attention, the importance of the cuticular (and behavioral) defenses remains under-examined. Knowledge concerning insect boundary defenses is vital to deciphering the factors that contribute to the virulence of entomopathogenic fungi. In this regard, although still limited, an increasing amount of research is suggesting that the cuticular surface is a major source for the adaptation of these fungi towards insect hosts and *vice versa*,* i.e.*, entomopathogenic fungi are an important source driving insect cuticular adaptations. Such information can also be used to define characteristics for understanding the complex interactions that can lead to failure of entomopathogenic fungi as biocontrol agents and the often seen discrepancies between laboratory and field experiments, hopefully leading to new insights aimed towards increasing the efficacy of mycoinsecticides as part of Integrated Pest Managements (IPM) practices. 

## 2. Infection by Entomopathogenic Fungi

**Figure 1:**
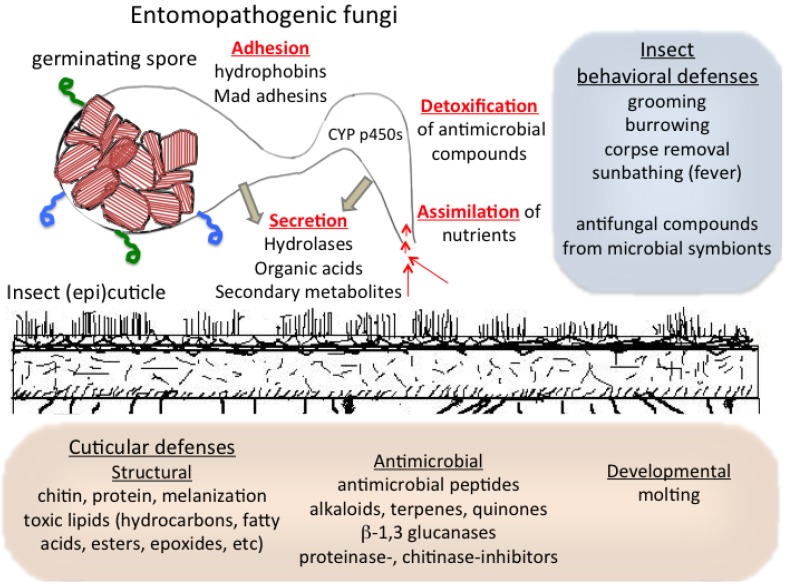
Overview of surface interactions between entomopathogenic fungi and the insect cuticle and host behaviors.

Unlike viruses and many nematodes and bacteria that require specialized routes of entry for infection of insect hosts, entomopathogenic fungi infect via penetration essentially anywhere on the host cuticle, although preferential sites have been noted on various insects. Infection begins with attachment of single-celled dispersive forms of the fungus, e.g., conidia or blastospores, to the insect cuticle (Figure 1). Expression of a variety of hydrolytic enzymes, e.g., proteases, chitinases, and lipases, and other factors, promote germination and growth of the fungus across the surface of the host, and subsequent penetration of cuticular layers [2,3]. During this process the fungus produces any number of specialized infection structures that can include penetration pegs and/or appressoria, which enable the growing hyphae to penetrate into the host integument. It is essentially in the later stages of this process that the pathogen encounters the host immune system. The insect cuticle itself is a highly heterogeneous structure that can vary greatly in composition even during the various life-stages of a particular insect. The epicuticle or outermost layer provides a hydrophobic barrier rich in lipids, and is followed by the procuticle that contains chitin and sclerotised protein, which is typically divided into the exo-, meso-, and endo-cuticular layers. The procuticle, in turn, is followed by the cells that constitute the epidermis, that sorrounds the internal structures of the insect.

Phylogenetic evidence suggests that fungal virulence towards insects has independently arisen several times, an example of convergent evolution [4]. The availability of genomic data has revealed that even among more closely related broad host range fungal pathogens of insects,* i.e.*, *Beauveria* (*Cordyceps*) *bassiana* and *Metarhizium* (*anisopliae*) *robertsii*, significant aspects of the molecular mechanisms that mediate virulence are dissimilar even though overall processes are similar [2]. Furthermore, unlike many plant pathogenic fungi, neither *B. bassiana* nor *M. robertsii* appear to have gene-for-gene avirulence mechanisms that determine host specificity, although homologs of effector-like proteins have been identified in the genomes of both fungi. Instead, as mentioned above, most current models suggest the primary mechanism of infection involves the production of a battery of hydrolytic enzymes that overwhelm the host in a degradative process that allows for penetration of fungal hyphae through the cuticle. Not too surprisingly, fungal enzymes that hydrolyze chitin and proteins (chitinases and proteases, respectively), which represent the major constituents of the insect cuticle, are considered vital to the infection process (Figure 1). Considerations on the coevolution of entomopathogen-derived hydrolytic enzymes and host defenses suggests diversification of fungal proteinases involved in virulence and expansion of host proteinase inhibitors of these enzymes due to reciprocal selection [5]. Numerous studies engineering entomopathogenic fungi to overexpress a variety of proteases, chitinases, and protease-chitinase fusion proteins have yielded strains displaying increased virulence as compared to their wild type parental isolates [6,7,8]. The importance of these enzymes is further highlighted by the diversity of genes (especially proteases) found in the genomes of these organisms. Although no systematic analysis of the activities of these enzymes have been performed to date, it is likely that some functional redundancy exists in their substrate specificity and that due to their mechanisms of action and targets that are found on essentially all insect hosts, these activities can be considered as broad-range virulence factors, and hence are unlikely to be required only for a specific subset of hosts. Intriguingly, where examined, chiefly as a consequence of the overexpression studies described above, the “natural” activities of these enzymes can be considered as sub-optimal,* i.e.*, if they were not rate limiting in some way overexpression should have had little to no effect. One potential reason for this is that although elevated activities of these proteins may lead to more rapid death, it also appears to lead to greater melanization or sclerotization of the host which results in a reduced ability of the infecting fungus to sporulate and disseminate from the cadaver, hence limiting the completion of the parasitic aspect of pathogen’s life cycle. Hydrolases, the mechanical pressure exerted by growing hyphae and/or appressoria are likely coupled to other factors, and the production of organic acids, particularly oxalate, by entomopathogenic fungi during infection may also contribute to weakening of cuticular structures including lipid breakdown [9,10].

## 3. Interactions with and Assimilation of the Host Cuticle by Entomopathogenic Fungi

Prior to reaching and degrading the chitin- and proteinaceous components of the insect, two other processes must occur; namely adhesion to and interaction with the epicuticular layer of the host (Figure 1). This outermost part of the cuticle consists of a thin (1–5 µm) deposition that constitutes the cement and waxy layers, which are comprised of a diverse array of lipids and other compounds [11,12]. Cuticular lipids and waxes, that often include abundant amounts of hydrocarbons, display considerable variation in content and composition between insects, and often within the different life stages/instars of each insect. Although information is scarce, due to the diversity and alterations in cuticular composition that can occur with insects (especially between larvae and adults), it is intriguing to speculate that the strategies employed by entomopathogenic fungi for infection may differ depending upon the life stage of the host. Nevertheless, adhesion to the epicuticular layer has been suggested to involve two steps, a non-specific passive adsorption of fungal cells on the surface of the host followed by consolidation of the attachment, although the distinction between these two processes is not clear [13,14]. Studies examining adhesion of *B. bassiana* to surface substrata showed essentially immediate (tight) binding of conidia to hydrophobic surfaces, but weak (washable) binding to hydrophilic surfaces [15]. Both *B. bassiana* and *M. anisopliae* produce hydrophobic conidia that contain a surface rodlet layer comprised of proteins termed hydrophobins [16]. In *B. bassiana* at least two hydrophobins (Hyd1 and Hyd2) are responsible for rodlet layer assembly, contributing to cell surface hydrophobicity, adhesion to hydrophobic surfaces, and virulence [17,18]. In *M. anisopliae*, two adhesin genes, termed *Mad1* and *Mad2* have been characterized [19]. These proteins contain signal peptides, threonine-proline rich regions, implicated in mediating adhesion, and putative glycosylphosphatidylinositol float sites, which would localize the proteins to the plasma membrane. Loss of *Mad1* resulted in decreased adhesion to insect cuticle, but also reduced germination, blastospore production, and virulence, suggesting coupling of surface sensing/adhesion to diverse downstream processes. Critically, expression of *Mad1* in yeast allowed it to adhere to insect cuticle. In contrast, *Mad2* appears to be important for plant interactions/adhesion and did not have any effect on adhesion to insect cuticles. Homologs of the *Mad* genes have also been identified in the *B. bassiana* genome, although whether they mediate similar functions remains to be determined. Furthermore, the mechanism of adhesion and the molecular targets of the adhesins have yet to be characterized. Potentially consistent with the model of adhesion described above, it is tempting to conclude that hydrophobins account for the relatively non-specific (passive) adsorption step and that the *Mad* adhesins are responsible for the more target specific consolidation (active?) stage, however, further experimental confirmation is needed. In addition to proteins, the surface of these fungal cells contains various carbohydrate moieties [20], although their contribution to adhesion has yet to be investigated. 

As mentioned, the surface composition of the insect cement and waxy layers includes various amounts of hydrocarbons (alkanes, alkenes, and their methyl-branched derivatives), fatty acids and esters, alcohols, ketones, and aldehydes, with minor components including triacylglycerols, epoxides, and ethers, as well as tanned (cross-linked) proteins that impact and direct important aspects of environmental and behavioral interactions of the insect [21,22,23] (Figure 1). Epicuticular constituents protect insects from dessication, are involved in chemical communication (within and between insect species especially in social insects) and defense, and have been applied for chemotaxonomic characterization of insects. Cuticular hydrocarbon composition can have differential effects on entomopathogenic fungi. Both *B. bassiana* and *M. anosipliae* can grow on long chain alkanes (up to C_33_) as well as many fatty acids, alcohols, and triacylglycerides. However, especially for growth on some longer chain substrates, preinduction on shorter chain compounds appears necessary, indicating that higher chain alkanes may be degraded under specific conditions or growth on these substrates may be an adaptive response [24,25,26]. Insect hydrocarbons, including aliphatic and methyl branched alkanes have been shown to be growth substrates for *B. bassiana*, e.g., the major components of the larval epicuticle of the blood-sucking bug *Triatoma infestans* Klug, a vector of trypanosomal disease causing parasites, includes saturated C_29_, C_31_, and C_33_, which can be utilized by *B. bassiana* [27]. Growth on long chain alkanes, C_24_ and C_28_ resulted in fungal peroxisomal proliferation and the substrates were degraded by *B. bassiana* into free fatty acids, phospholipids, and acylglycerols [28,29]. The production of volatile organic compounds has also been linked to carbon growth source, with alkane grown cells producing *n*-decane as a by-product of β-oxidation reactions [28,29]. It should be noted that environmental factors, e.g., temperature, humidity, and sunlight, are likely to affect adhesion, germination, growth (and penetration) on insect cuticles and their components. 

Portions of the biochemical and genetic determinants of hydrocarbon assimilation pathways in entomopathogenic fungi have been elucidated. Use of radiolabeled hydrocarbons to probe the biochemical mechanisms of alkane catabolic pathways in *B. bassiana*, revealed a degradative pathway involving β-oxidation by cytochrome P450 enzyme systems. Oxidation by P450 enzymes is followed by successive transformations to yield fatty acyl CoAs, reactions occurring in peroxisomes and/or mitochondria [30,31]. Thus far, eight cytochrome P450 (CYP) genes, four catalases, three lipase/esterases, long chain alcohol and aldehyde dehydrogenases, and a putative hydrocarbon carrier protein have been implicated as potentially participating in cuticular lipid degradation in *B. bassiana* [25,32]. To date, the enzymatic activity and role of only one such protein, BbCYP52X1, has been confirmed as contributing to hydrocarbon assimilation and virulence via characterization of a targeted insertion mutant of the gene and heterologous expression and characterization of the protein in a yeast expression system [24]. Consistent with its predicted role, the *ΔBbCYP52X1* strain was impaired in virulence when applied on the cuticles of insect hosts, but not when injected (*i.e.*, when the cuticle was bypassed). Targeted gene‑knockouts of the other CYP genes had no effect on virulence, suggesting either that these proteins do not play a role in cuticular hydrocarbon assimilation or that functional redundancy masked any phenotypes [32]. The latter has precedence in that the alkane assimilating yeast *Yarrowia lipolytica* contains a similar diversification of hydrocarbon utilizing CYP genes and that single gene knockout mutant strains do not show any significant phenotype, with disruption of several genes required in order to lose the ability to utilize certain (hydrocarbon) substrates [33]. With respect to the catalases and lipases, catalase mutants, although available [34], have yet to be tested for any impairment in hydrocarbon utilization and lipase/esterase mutants have yet to be reported. However, modulation of secreted lipolytic activity in response to host integument components and various fatty acids and esters has been observed in *M. anisopliae*, although cuticular specific extracts were not examined [35]. Pre-induction or adaptation on media containing hydrocarbons, specifically use of fungal conidia harvested from alkane containing media, results in more virulent spores. *B. bassiana* cells grown on hydrocarbons display a 2–4 fold increase in mortality against the bean weevil *Acanthoscelides obtectus* (Say), when compared to cells grown on glucose [36]. It is likely, therefore, that recognition of cuticular lipids whose assimilation represents important metabolic adaptations, activate signaling cues that contribute to entomopathogenesis. In addition, the process of fungal growth on the host surface results in alterations of the hydrocarbon contents of the cuticle [37,38]. 

## 4. Cuticular Mechanisms of Host Resistance to Microbes: Structural and Chemical

Cuticular lipids, however, can also promote or inhibit fungal attachment to cuticle. Such attachment can be affected by nutritional requirements and can be enhanced via formulation that can result in increased efficacy of the fungal agents against target insects [14,39,40]. Entomopathogenic fungi transformed with green fluorescent protein (gfp) have been used to examine adhesion to certain insects, although high endogenous autofluorescence from the cuticular surfaces of many insects may limit quantification of adhesion using such an approach [41]. The hydrophobic nature of the epicuticle is generally considered a good substratum for adhesion of fungal spores. However, specific adaptations have been reported; e.g., the cuticular fatty amides of the booklouse, *Liposcelis bostrychophila* L., appear to prevent adhesion of (dry) conidia of entomopathogenic fungi to the insect [42]. Although limiting adhesion to the cuticle may be a rather rare defense mechanism, it is well known that differences in the hydrocarbon content of the waxy layer can have profound effects on fungal pathogenesis and a variety of antimicrobial compounds are secreted to the cuticular surface. Cuticular lipids and aldehydes from the Southern stink bug, *Nezara viridula* L. have a fungistatic effect on *M. anisopliae*, and cuticular extracts from *Heliothis zea* Boddie display toxicity towards *B. bassiana* [43,44]*.* The brassy willow leaf beetle, *Phratora vitellinae* (L.), releases volatile glandular secretions, with salicylaldehyde identified as the major component of their enveloping perfume cloud, that exhibit toxicity against entomopathogenic fungi, to help sanitize their microhabits [45]. Similarly, larvae and adults of the mustard leaf beetle, *Phaedon cochleariae* (Fabricius), exude glandular secretions, containing the iridoid monoterpene epi-chrysomelidial, which display antifungal activity against *B. bassiana* [46]. Free fatty acids on the surfaces of various Lepidoptera species and fatty acids isolated from the biting midge, *Forcipomyia nigra* Winnertz, were able to inhibit germination of a range of entomopathogenic fungi [44,47,48]. Inhibition, however, is often demonstrated* in vitro* using high concentrations of (cuticular) extracts and in most cases the test organism remains susceptible (to varying degrees) to infection. Such results indicate that the concentrations found on individual insects may be effective against fungi in general but are not high enough to ward-off entomopathogenic fungi and/or that entomopathogneic fungi have evolved detoxifying systems for overcoming (some) insect antifungal compounds. In this regards, cuticular pentane extracts derived from the European common cockchafer (*Melolontha melolontha* L.) inhibited spore germination and hyphal growth when tested against a non-pathogenic strain of *B. bassiana* but had no effect against a pathogenic strain [49], and cuticular extracts of the pea aphid differentially effected germination and production of penetrant structures of aggressive and non-aggressive strains of the entomopathogenic fungus *Conidiobolus obscurus* [50,51]. The production of antimicrobial compounds can extend to unusual aspects of insect biology. Burying beetles (genus *Nicophorus*) use (bury) small vertebrate carcasses as reproduction and breeding sites that are protected from microbial decay via the production of a myriad of antibacterial and antifungal compounds, which are effective at suppressing entomopathogenic fungi [52]. Both *M. anisopliae* and *B. bassiana* are able to infect across Arthropoda classes, and pentane extracts of two closely related tick species, one highly susceptible to *B. bassiana* (*Amblyomma maculatum* Koch) and the other somewhat resistant to fungal infection (*A. americanum* L.), were differentially able to inhibit fungal germination, with good growth seen in media supplemented with extracts from the former tick species and poor fungal growth observed in the presence of extracts from the latter species [53]. Similarly, *Metarhizium* strains infective towards the cattle tick *Rhipicephalus annulatus* Neumann were able to germinate, grow on, and penetrate the susceptible tick species cuticle, whereas fungal conidia were able to germinate but could not sustain hyphal growth on two resistant tick species, *Hyalomma excavatum* Koch and *R. sanguineus* Latreille [54]. As methods for analysis of the lipid composition of the insect cuticle improve, the identification of additional (bio-)fungicides is likely to occur [55]. 

In addition to cuticular lipids, surface antifungal defenses include small molecule toxins (including peptides) and proteins. While it remains unclear whether any small compound antifungal peptides are actually secreted to the insect surface, a number of insect-derived chitinase and protease inhibitors as well as a wide range of antimicrobial peptides (AMPs), some of which demonstrate the hallmarks of co-evolutionary pressures, have been reported [5]. Fire ant (*Solenopsis invicta* Buren) venom alkaloids display aseptic activity and can inhibit germination and development of a number of entomopathogenic fungi [56], although protection by alkaloids is not universal [57]. Subterranean termites produce defensin-like antifungal peptides known as termicins, but have also been reported to secrete antifungal β-1,3-glucanases (also termed GNBPs, Gram-negative bacteria binding proteins) to the cuticle [58]. Inhibition of the glucanase activity using D-δ-gluconolactone or transcriptional repression of the gene via RNA interference has been shown to result in increased susceptibility of termites to *M. anisopliae* [59,60]. Certain tenebrionid species, e.g., the beetle *Ulomoides dermestoides* Fairm, produce cuticular benzoquinones that have been shown to inhibit fungal growth and may help account for the relatively high resistance seen in these insects to infection by entomopathogenic fungi [61]. Cuticular phenolic compounds coupled to the activity of tyrosinases results in melanization, often as a response to microbial attack. Melanin and melanized tissues may be directly toxic to microbes inhibiting germination and/or hyphal growth, can be more resistant to mechanical penetration and/or the action of fungal degradative enzymes, and can act as a barrier to limit nutrient uptake by the pathogen [62]. Protease activity on the part of the fungus, however, has been shown to contribute to solubilization of cuticle-bound melanin, and chymoelastase (Pr1) in *M. anisopliae* is resistant to inhibition by melanizing mixtures [62]. Melanic *Spodoptera littoralis* Boisduval larvae were more resistant than their amelanic counterparts to *B. bassiana* infection, supporting a role for this process in microbial defense against fungi; however, in *S. exempta* Walker no significant association between fungal susceptibility and melanism was seen, indicating the complex nature of the interaction between microbial pathogens and host defenses and the variability in outcomes that can occur [63]. 

## 5. Cuticular Developmental, Symbiotic, and Behavioral Defenses against Microbes

Simple molting may provide a means for avoiding infection. Rapid ecdyces in aphids may be an important contributing factor to poor outcomes in applications of entomogenous fungi and molting in the diamondback moth (*Plutella xylostella* L.) may also help minimize the ability of fungal pathogens to fully infect the host [64,65]. In this regards, protease inhibitors highly inhibitory towards *M. anisopliae* Pr1 protease have been detected in the molting fluid of the tobacco hornworm, *Manduca sexta* L. [66]. However, molting within 24 h of *Metarhizium* spp. inoculation had no significant effect on subsequent mortality in the acridid pests *Chortoicetes terminifera* Walker (locust) and *Phaulacridium vittatum* Sjöstedt (wingless grasshopper) [67]. In addition, some entomopathogenic fungi appear to inhibit molting of their hosts via oxidative inactivation of host ecdysteroid [68].

Aside from the cuticle and any insect (surface) defensive compounds and proteins, the potential role and importance of exogenous and endogenous (symbiotic?) microbial communities in defending against microbial (fungal) pathogens is gaining increased interest [69]. Since some insects have obligatory symbionts that provide the host with required nutrients, e.g., essential amino acids, co‑factors and vitamins, determining any contributions to microbial defense can be difficult. Some facultative symbionts manipulate host biology and behavior including feeding, reproduction, and homeostatis, and thus examining their potential protective effects against natural enemies can run into confounding effects,* i.e.*, experiments eliminating such bacteria via antibiotic treatment may result in pleiotropic effects or may simply not be feasible. Regardless, the role of exo/endo-genous microbes in contributing to host defense against entomopathogenic fungi has been reported. *Drosophila* bearing the endosymbiotic bacterium *Wolbachia* appear to display a generalized resistance to insect pathogens including *B. bassiana* [70]. Symbiotic bacteria from antibiotic producing *Streptomyces* species have been implicated in protecting larvae of a solitary hunting wasp (European beewolf, *Philanthus trianglum* Fabr.) from fungal infection [71]. The Southern chinch bug, *Blissus insularis* Barber have midgut crypts that contain high densities of Burkholderia bacterial species that are known to produce potent antifungal compounds and may help account for enhanced resistance that these insects often display towards entomopathogenic fungi [72]. Several ant species cultivate actinobacteria, again prokaryotes, that produce potent antimicrobial compounds, not only potentially as a means of protecting conspecifics and brood from microbial pathogens, but also, in the case of fungus growing ants, to protect their gardens from other parasitic fungi [73,74].

Finally, considerations of the interactions between entomopathogenic fungi and the cuticle surface can elicit specific behavioral responses (often in conjunction with chemical/proteinaceous defenses and/or the actions/products of symbiotic bacteria) in the insect meant to limit the ability of the pathogen to parasitize the host (Figure 1). For social insects, nestmate grooming is a well‑recognized mechanism for minimizing potentially harmful microbes, and for other insects self‑grooming may likely play a similar role. Ants disinfect fungus-exposed brood via a combination of (allo-) grooming and disinfection using formic acid and other chemical compounds [75]. Mutual grooming in a number of termite species represents an effective means for eliminating potential fungal infections mediated by either *M. anisopliae* or *B. bassiana* [76,77]. Termites display odor aversion to pathogenic fungi, with more virulent strains either capable of masking odors or produce less volatiles that can be detected by the insect [78]. Termite antennae have been shown to play an important role in both fungal odor detection and grooming [79]. Taken together; grooming, cuticular defense responses including encapsulation and termicin production, combined with β-1,3 glucanase activity and elevated colony temperatures, provide a formidable and potentially synergistic barrier to infection by entomopathogenic fungi [80], and highlight the importance of defending against these pathogens in the biology and evolution of termites. Temperature elevation whether via intrinsic biological mechanisms or by behavioral adaptions, can also help suppress infections [81]. In the latter case, the phenomenon known as “behavioral fever”, can be achieved by aggregation as seen in some ant and termite colonies or by heat seeking such as sunbathing as has been reported for various acridids [82,83,84]. Aside from temperature effects, gregarious locusts appear to have an advantage in resistance to *M. anisopliae* than their solitary counterparts [85]. In an interesting multi-faceted response, the western boxelder bug (*Boisea rubrolineata* Barber), form aggregations in warm sunlight that stimulates release of monoterpenes that can inhibit germination of *B. bassiana* conidia [86]. Sunbathing can not only act through temperature elevation but also via exposure to UV irradiation that can limit fungal growth [87,88]. Corpse and removal of chronically infected nestmates can also decreased the spread of contagions [89], particularly decreasing the effectiveness of using entomopathogenic fungi for control of social insects. Simple burrowing into the soil of infected larvae can result in removal of the fungal spores [90]. It should be noted, however, that some of these behaviors, e.g., corpse removal, are not always or only limited to the cuticle surface, in contrast to for example, grooming. In a combination of behavior and chemical adaptation, some insect target hosts show a positive density dependent alteration in the degree of melanization of the cuticle in response to perceived increased risk of disease due to infection of conspecifics [91]. In terms of biological control applications, some of these behavioral responses can be overcome by the addition of low concentrations of insecticides. Sub-lethal concentrations of imidacloprid, when combined with entomopathogenic fungi, can overcome the behavioral defenses of leaf cutter ants (*Acromyrmex subterraneus* Ferel), the burrowing behavior of larvae of the citrus root weevil (*Diaprepes abbreviates* L.), and even increase effectiveness of the fungi against the mosquito dengue vector *Aedes aegypti* L. [92,93,94]. Entomopathogenic fungi can also be engineered to express molecules that would affect insect behavior, thus potentially increasing their effectiveness and more thermotolerant strains can be selected for [95,96]. In addition, formulation strategies including use of pathogen combinations, UV‑absorbing dyes, and sublethal doses of insecticides can mitigate the deleterious consequences of certain behavioral defenses [97,98,99,100].

## 6. Conclusions

Insects have evolved a formidable array of defenses against microbes that are ubiquitous in the environments that they inhabit. These extend from the cuticle that can be reinforced with antimicrobial compounds and secretions to behavioral adaptions including induced fever, grooming, and burrowing to developmental programs of molting which effectively results in cleansing of the outer surface of the insect to the recruitment of antibiotic or other defense compound producing (symbiotic) bacteria. Furthermore, although not considered here, the potential role of plants (and plant derived compounds) in assisting either the (fungal) pathogen or the insect host cannot be ignored. Within this context, *i.e.*, excluding viruses and bacteria that enter *per os*, it is possible to suggest that the humoral response represents a last ditch effort to thwart fungal pathogens or else deal with small inocula. In response, entomopathogenic fungi must breach the cuticle, detoxify host and/or endogenous microbial defenses, evade grooming and other behavioral responses, and potentially suppress other pathogens and parasites. Regarding virulence, two competing pressures are likely to be exerted on the fungus: (a) to specialize on specific (abundant) target hosts; and/or (b) to maintain broad host range. Fungal adaptive responses may be mediated by epigenetic mechanisms that would allow for short-term specialization while maintaining the broad host range potential. Current evidence is increasingly suggesting that a major factor driving the co-evolutionary arms race between the pathogen and the host occurs on the cuticular surface, and although significant progress has been made in recent years, much regarding the molecular determinants that mediate these interactions in both the pathogen and the host remains to be uncovered. Further research examining genetic variation regarding cuticular degradative processes amongst hypervirulent fungal strains on the one hand, and cuticular defense responses in resistant insect species on the other, is warranted. If the “action on the surface” acts as a major force in the adaptive interplay between the host and the pathogen, one could predict that enhanced resistance can be found in natural populations via several potential mechanisms that can include: (i) enhanced or alerted cuticular lipid composition; (ii) increased melanization and/or sclerotinization of the cuticle; (iii) more rapid development (ecdysis); (iv) increased detection and avoidance behaviors; and (v) recruitment/selection of symbionts that produce antifungal compounds. Conversely, adaptive responses on the part of the pathogen could include: (i) more rapid germination and penetration; (ii) increased resistance to antifungal compounds; and (iii) increased production of secondary metabolites that target not only the host, but competing microbes. Knowledge concerning these processes can be exploited in rational design strategies for improving the effectiveness of entomopathogenic fungi in field applications. 
